# The Health Risks of Electronic Cigarette Use to Bystanders

**DOI:** 10.3390/ijerph16091525

**Published:** 2019-04-30

**Authors:** Wouter F. Visser, Walther N. Klerx, Hans W. J. M. Cremers, Ramon Ramlal, Paul L. Schwillens, Reinskje Talhout

**Affiliations:** National Institute for Public Health and the Environment (RIVM), Centre for Health Protection, Anthonie van Leeuwenhoeklaan 9, 3721 MA Bilthoven, The Netherlands; wouter.visser@rivm.nl (W.F.V.); walther.klerx@rivm.nl (W.N.K.); hans.cremers@rivm.nl (H.W.J.M.C.); Ramon.ramlal@rivm.nl (R.R.); Paul.schwillens@rivm.nl (P.L.S.)

**Keywords:** electronic cigarettes, bystanders, health risks, second hand vaping

## Abstract

This works aimed to assess the health risks of e-cigarette use to bystanders. The exhaled breath of 17 volunteers was collected while they were vaping, and the levels of nicotine, propylene glycol, glycerol, formaldehyde, acetaldehyde, acrolein, tobacco-specific nitrosamines (TSNAs), and heavy metals were analyzed. Increased levels of nicotine, propylene glycol, TSNAs and copper were found in the exhaled breath of the volunteers. From these measurements, bystander exposure was estimated for two different scenarios: (1) A non-ventilated car with two e-cigarette users and (2) a ventilated office with one e-cigarette user. Our results show that bystanders may experience irritation of the respiratory tract as a result of exposure to propylene glycol and glycerol. Systemic effects of nicotine should also be expected if nicotine-containing e-liquid is used, including palpitations, and an increase of the systolic blood pressure. Furthermore, due to the presence of TSNAs in some e-liquids, an increased risk of tumors could not be excluded for the ‘car’ scenario. While e-cigarette use can clearly have effects on the health of bystanders, the risks depend on the rate of ventilation, dimensions of the room, and vaping behavior of the e-cigarette user. The presence of TSNAs in e-liquids can be avoided, which will prevent the most serious effect identified (increased risk of tumors).

## 1. Introduction

With the increased popularity of electronic cigarettes, concern about the health risks associated with these devices has grown. Most studies to date indicate that e-cigarettes are less harmful than tobacco cigarettes [1], but much uncertainty remains regarding the absolute health risks of e-cigarette use, especially with respect to long-term effects and the risks of inhaling flavor components and thermal decomposition products. Furthermore, the technical design of e-cigarettes and their operation continues to evolve rapidly, demanding continuous adaptation of research methods.

Most e-cigarette health risk studies have focused on the risks to users. Comparatively little is still known regarding the risks of exposure to second-hand e-cigarette emissions, to bystanders. Different approaches have been described to estimate the exposure of bystanders, reviewed by Fernandez et al. [2] and Abidin et al. [3]. Some estimates have been based on a chemical analysis of machine-generated e-cigarette vapor. However, this approach does not account for retention in the respiratory system of the e-cigarette user, resulting in an overestimation of the exposure.

Others have simply assumed that bystander exposure to nicotine from e-cigarettes is similar to that of tobacco cigarettes [4]. However, because as much as 85% of the nicotine in environmental tobacco smoke originates from side-stream-smoke [5], this also results in an important overestimation of nicotine exposure. Furthermore, emissions other than nicotine were not considered. Ballbé et al. [6] estimated bystander exposure by sampling surfaces and air in the residences of e-cigarette users, and from any biomarkers of exposure in individuals that share residence with e-cigarette users. They found it difficult to exclude or account for the exposure of the test subjects to tobacco smoke elsewhere.

A better approach is to analyze the air in a room in which volunteers use e-cigarettes [7,8]. This method can provide an indication of the actual exposure level. However, it is technically challenging to measure e-cigarette emissions after they have become diluted with a large volume of air in a test room. Therefore, in this work, the exhaled breath of volunteers was sampled directly while they were vaping. The concentrations of nicotine, propylene glycol, glycerol, formaldehyde, acetaldehyde, tobacco-specific nitrosamines and heavy metals were measured, because these compounds were previously found to contribute to the health risk to users of e-cigarettes [9], including irritation and damage of the respiratory tract, increased blood pressure, palpitations, and an increased risk of cancer. The emission in the exhaled breath were then used to assess the health risk to the bystanders for two different scenarios.

## 2. Materials and Methods

### 2.1. Recruitment of Test Subjects

The study was reviewed and approved by the Medical Research and Ethics Committee of Wageningen University (registration code NL53471.081.15). E-cigarette users meeting the following inclusion criteria were initially identified by Computer Assisted Web Interviewing (CAWI): (i) At least 18 years of age, (ii) daily e-cigarette use, with a nicotine-containing (>6 mg/mL) liquid (iii) no clinical diagnosis of diabetes or lung disease. Gender and ‘dual use’ status were also recorded. 44,439 respondents from a national database maintained by TNS-NIPO (http://www.tns-nipo.com) were queried, identifying 113 potential test subjects. The volunteers for our study were recruited from this group, matching the gender ratio and dual-use status to that which was observed in the CAWI.

### 2.2. Collection and Analysis of Exhaled Vapor

Three e-cigarette/e-liquid combinations were used (Table 1). Subjects took a specified number of puffs and exhaled onto a trapping device immediately after each puff via a mouthpiece. The trapping device contained a quartz fiber pad (42 mm Cambridge Filter Pads (CFP), Borgwaldt, Germany) and a carboxen-572 cartridge (Sigma-Aldrich, Zwijndrecht, The Netherlands) for the analysis of aldehydes (discussed below). The trapping devices and e-cigarettes were weighed before and after each experiment. The flowrate and volume of exhalation into the trapping devices was measured with a small flowmeter (TSI4000, TSI Inc., Shoreview, MN, USA). Immediately after collecting the last exhalation, the filter holders were weighed, and the components of interest were extracted and analyzed. Samples of control breath (without using the e-cigarette) were obtained from each subject at the start of the experiment (i.e., before taking the first puff from an e-cigarette). Subjects did not smoke nor vape for at least 30 min prior to the start of the experiment. Nicotine, propylene glycol and glycerol could be measured with the material collected from five puffs, and were measured for all test subjects. For the other chemicals (tobacco-specific nitrosamines (TSNAs), aldehydes and metals), 25 puffs were collected. To limit the number of puffs for each volunteer, only one of these analyses was performed for each volunteer.

### 2.3. Nicotine, Propylene Glycol and Glycerol

CFP filters were used to trap propylene glycol, glycerol and nicotine. Per subject, five exhalations were collected onto a single filter. The analytes were extracted from the filters with methanol containing 1,3-butanediol and heptadecane as internal standards for humectants and nicotine, respectively. Propylene glycol and glycerol were analyzed in accordance with the World Heath Organization (WHO) TobLabNet SOP6 [10]. Nicotine content was determined with LC-MSMS (AB SCIEX, Nieuwerkerk aan den Ijssel, the Netherlands). Calibration curves were prepared according to TobLabNet SOP6 and ISO-10315 for PG/glycerol and nicotine respectively, and measured before and after each analytical run. Repeatability for all three analytes is <10% RSD. Recovery of nicotine is 85–115%, recovery of propylene glycol and glycerol is 90–110%.

### 2.4. Tobacco-Specific Nitrosamines (TSNAs)

TSNAs were collected on CFPs. Per subject, 25 exhalations were collected on a single filter. Stable-isotope labeled NNN-d_4_, NNK-d_4_, NAB-d_4_ and NAT-d_4_ (Toronto Res. Chem., Toronto, ON, Canada) were added to the filter as internal standards. The TSNAs were extracted by the addition of 5 mL of 10 mM NaOH solution, and 10 mL of methyl-tert-butylether (MTBE). After 30 min of gentle shaking at room temperature the MTBE extract was removed. A second extraction with 10 mL MTBE was performed and combined with the first extract. The extract was evaporated to dryness under nitrogen. The residue was dissolved in 0.1% formic acid and analyzed with LC-MSMS. Repeatability is <5% RSD, recovery is 85–115%.

### 2.5. Carbonyls

Carbonyls were collected using a combination of a carboxen-572 (CX572) cartridge and a CFP, as described by Uchiyama et al. [11], with the following modifications. To reduce the restriction of airflow by the CX572 cartridge, the plastic-fritted disks at each end were replaced by fine mesh stainless steel screen. After sample collection, the CX572 beads from the cartridge and the filter were transferred to a stoppered flask, and 10 mL of a mixture of methanol and carbon disulfide (80:20 v/v) was added. After 20 min of shaking at room temperature, a 0.5 mL sample of the extract was derivatized with dinitrophenylhydrazine (DNPH), and processed as described by Uchiyama et al. [11]. In parallel with each sample, a corresponding blank sample of the methanol/carbon disulfide solvent mixture was analyzed and used to correct for the small amount of contaminating aldehydes that were present in the solvent mixture. A calibration curve was prepared using a mixture of pre-derivatized carbonyl-DNPH analytical standards (Sigma-Aldrich, Zwijndrecht, The Netherlands). Per subject, 25 exhalations were collected onto each filter for this analysis (i.e., subjects took 25 puffs, and after each puff exhaled their first exhalation onto the filter). Repeatability for formaldehyde is <10% RSD, and <10% for acetaldehyde and acrolein. Recovery for formaldehyde is 80–120%, for acetaldehyde and acrolein 90–110%.

### 2.6. Metals

Whatman 47 mm QM-A grade filters (Whatman, Maidstone, UK) were used to collect samples for the analysis of metals. Per subject, 25 exhalations were collected onto each filter. The samples were digested using a 12:1 (v/v) mixture of 65% (w/v) nitric acid and 30% (w/v) hydrogen peroxide. After digestion, the samples were analyzed using inductively coupled plasma mass spectrometry (ICP-MS). Blank filters were analyzed in parallel to correct for the metal background content of the filters. Repeatability for all metals is <10% RSD.

### 2.7. Estimation of Bystander Exposure

One exhalation following each puff was collected for analysis. This measurement was used to estimate the total amount of chemicals exhaled. For this purpose, the total amount of each chemical inhaled by the e-cigarette user (A_puff_) was first calculated, using a model originally developed for cigarette smoking [12]. This calculation is included in Supplementary Material 2 (in the section titled ‘Estimation of the total amount of a chemical exhaled by a vaper following one puff’). Worst-case assumptions were made with regard to alveolar retention in the calculation of the fraction of each chemical that is exhaled in the first breath. As explained in detail in Supplementary Materials 2, this requires different assumptions for the evaluation of local pulmonary and systemic effects. The first exhalation was calculated to contain respectively 40% (for local pulmonary effects) and 33% (systemic effects) of the total amount of chemical that was inhaled. The maximal final air concentration (mg/m^3^) of each chemical to which bystanders are exposed in the different scenarios was then calculated as follows:

conc = (1 − F_pulm,ret_) × A_puff_ × f × t × n/V (1)

F_pulm,ret_ = pulmonary retention fraction (zero or 0.5 for local and systemic effects, respectively) (2)

A_puff_ = amount of a chemical inhaled with one puff (mg) (3)

f = puff frequency (min − 1)(4)

t = vaping period (min)(5)

n = number of persons vaping(6)

V = volume of room (m^3^) (7)

ConsExpo [13] was used to determine the effect of ventilation in the ‘office’ scenario. The final concentration in the ‘office’ scenario after four hours was reduced to 42.5% of the value without ventilation.

### 2.8. Risk Assesment

To evaluate local effects on the respiratory tract and systemic effects, respectively, the air concentration (final concentration (mg/m^3^) reached at the end of the vaping period) and internal systemic exposure (expressed as mg/kg bw), were used. For each chemical, the exposure concentrations were calculated from the highest amounts exhaled by the volunteers. For the four nitrosamines, the total NDMA equivalent was calculated for each volunteer, and the highest total NDMA equivalent was used. The estimated air concentrations for the individual chemicals were compared with human limit values with respect to chronic exposure for the general population (Supplementary Material 1). Air Quality Guidelines as derived by the WHO were used as the first choice [14]. Air concentrations of chemicals below their limit value are considered not to result in adverse health effects. In cases where appropriate human health-based limit values were lacking, the risk assessment was based on a Margin of Exposure (MOE) approach. The evaluation of the MOE accounted for differences in sensitivity between animals and humans if applicable, and between human individuals and differences in exposure pattern between that for the point of departure (PoD) and the bystander. 

With respect to carcinogens, for which no safe threshold can be derived, an MOE of at least 10,000 (relative to a BMDL10 value, also see Supplementary Material 2) was considered sufficient to support the conclusion that the exposure scenario for that chemical is of ‘low concern’, i.e., the risk for tumors is then considered to be very low following the European Food Safety Authority (EFSA) approach for food products [15].

## 3. Results

### 3.1. Recruitment of Volunteers

**Figure 1:**
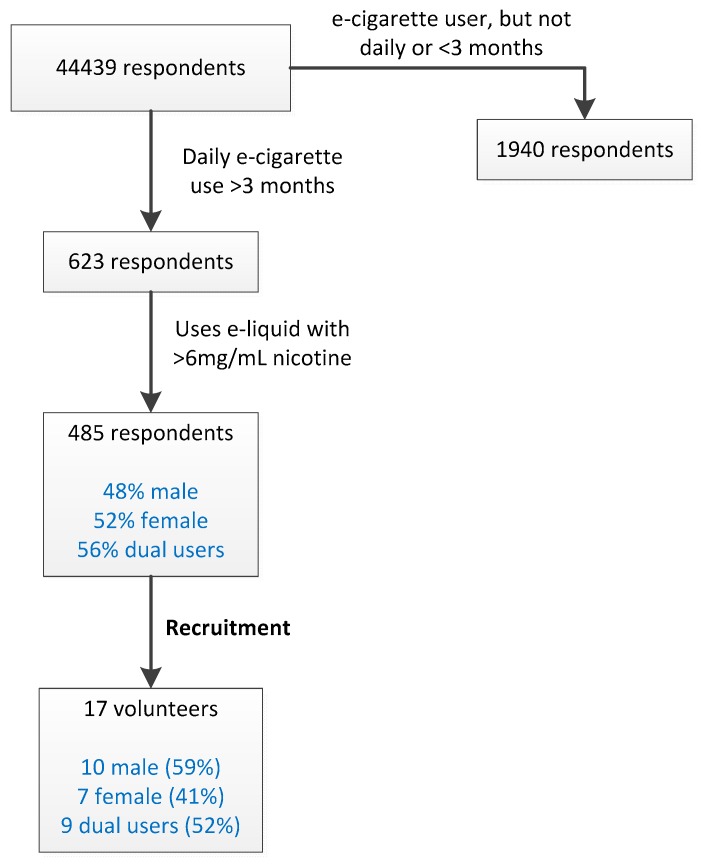
Screening and recruitment of volunteers.

By CIWA screening, 485 e-cigarette users were identified that matched the inclusion criteria (Figure 1). Volunteers were recruited from this group. The gender ratio and percentage of dual users were approximately matched to those observed in the group of 485 daily e-cigarette users (Figure 1). 17 volunteers participated in the vaping experiment: 10 male and 7 female. Nine participants were dual users.

### 3.2. Vaping Experiment

E-cigarettes were weighed before and after the experiment. E-liquid consumption per puff was 5.6 ± 2.4 mg (average ± SD). 4 participants used e-cigarette/liquid combination A (Table 1), 6 used B and 7 used C.

### 3.3. Analysis of Exhaled Vapor

The trapping devices were weighed before and after each vaping session (Table 2).

The mass gain of the trapping devices was significantly higher for exhaled e-cigarette vapor (4.24 ± 2.54 mg per exhalation) than for control breath (1.86 ± 1.28 mg per exhalation). An analysis of the water content of the methanol extracts of the filters revealed that the mass gain and water content of the filters are strongly correlated (R = 0.98), indicating that water constitutes most of the mass gained.

A summary of the chemical analysis of the exhaled vapor is shown in Table 3. The exhaled volume exhibited considerable variation between subjects (ranging from average volumes of 33 mL to 1528 mL per exhalation). Nicotine was detected in all samples except one. The control breath samples also contained small amounts of nicotine (up to 0.2 ng per puff) likely due to (e-)cigarette use and second-hand exposure prior to the experiment. For all chemicals, the amounts observed in the control samples were subtracted from the amounts measured in breath exhaled during e-cigarette use.

Propylene glycol was observed in the exhaled breath from 4 out of 17 subjects, but glycerol remained below the limit of quantification. While the e-liquids used do contain glycerol, its concentration was 2- to 4-fold lower than that of propylene glycol. Additionally, the sensitivity of the analytical method was lower for glycerol, mainly because the chromatographic peak is wider.

The four TSNAs are listed individually, as well as total TSNAs exhaled, expressed as NDMA equivalent (calculated for each volunteer individually). There was no significant difference in total TSNAs between dual users and exclusive e-cigarette users (student’s *t*-test, *p* = 0.79)

Aldehydes in exhaled breath during e-cigarette use were below the limit of quantification in all four samples. E-cigarettes are known to generate formaldehyde and acetaldehyde during normal use [9,16], but these chemicals are very reactive and water-soluble. They will be efficiently absorbed in the environment of the human respiratory tract. While formaldehyde, acetaldehyde and acetone were detected, the amounts observed in exhaled vapor did not exceed the levels in control breath. It is well established that small amounts of aldehydes and ketones, including formaldehyde, acetaldehyde and acetone occur naturally in exhaled breath

### 3.4. Bystander Exposure

Two specific scenarios were evaluated. The first scenario concerns a daily car trip of one hour in a small unventilated car with two e-cigarette users. The bystander is a child, sitting in the same car. This exposure scenario approximates the highest levels of exposure that may be expected in everyday situations. The second scenario concerns a daily exposure of four hours in an office-sized space with one e-cigarette user. The parameters defining the two scenarios are listed in Table 4. Exposure estimates for the two scenarios are listed in Table 5.

### 3.5. Risk Assessment

It should be noted that the MOE was calculated from the final (highest) concentration reached by each chemical after a vaping session, whereas the level of chemicals in the air actually builds up gradually. This overestimate of bystander exposure was taken into consideration in the evaluation of the MOEs below. Furthermore, interindividual variability in sensitivity between bystanders exists, and was also considered in the assessment.

### 3.6. Glycerol and Aldehydes

Glycerol, formaldehyde, acrolein and acetaldehyde were not detected. Based on the available toxicological information, it was concluded that amounts of these chemicals below the limit of quantification (LOQ) are not expected to induce adverse health effects.

### 3.7. Propylene Glycol

To evaluate the risks of propylene glycol, we have to consider that (i) exposure to second-hand e-cigarette vapor is less-than-lifetime, (ii) we extrapolate from a rat-based study to effects in humans, (iii) there is inter-individual variability in sensitivity among bystanders and (iv) differences exist between the exposure profile in the animal studies and the (daily) e-cigarette bystander exposure.

Furthermore, the retention of PG is unknown, and the default value for the retention that was assumed constitutes a worst-case systemic exposure estimate. Based on the MOE (Table 6) and above considerations, we do not expect systemic effects to occur upon exposure to propylene glycol for bystanders of e-cigarette vaping. Local effects on the respiratory tract and eyes cannot be excluded for the ‘car’ scenario. However, it is expected that effects are meant to be mild, if they occur. For the ‘office’ scenario, no local effects on the respiratory tract are anticipated.

### 3.8. Nicotine

An appropriate PoD for evaluating a lifetime inhalation exposure is currently not available, prohibiting an MOE approach. A weight-of-evidence evaluation was therefore applied.

### 3.9. Nicotine: Local Effects on the Respiratory Tract

The exposure concentration of both scenarios are below the No-Observed-Adverse-Effect Level (NOAEL) by a factor 3–14 described in a two-year rat inhalation study [17,18].

Furthermore, the exposure concentrations for the ‘car’ and ‘office’ scenarios are approximately a factor of 170 and a factor of 750, respectively, below the effect level in a study in which human volunteers inhaled nicotine, while cough response and airway constriction were monitored. Taking into account differences in exposure profile between the human study on the one hand and the (daily) exposure of the bystander on the other hand, local effects on the respiratory tract upon exposure to nicotine for a bystander of e-cigarette vaping are not expected for either scenario.

### 3.10. Nicotine: Systemic Effects

Only a small margin exists between our estimated bystander exposure and the effect level for systemic effects described in the human study described in [17,19]: Respectively, 2.1 and 6 for the ‘car’ and ‘office’ scenarios. Especially considering differences in exposure duration and the fact that effects were observed at the PoD. Also taking into account the interindividual variability in sensitivity among bystanders, systemic effects (increased heart rate and increased systolic blood pressure) should be expected for the ‘car’ scenario as a result of the nicotine exposure. For the ‘office’ scenario, it cannot be excluded that such systemic effects occur. The magnitude of the increased heart rate and increased systolic blood pressure are comparable to what may be expected from the intake of the amount of caffeine contained in 2 or 3 cups of coffee.

### 3.11. Tobacco-Specific Nitrosamines (TSNAs)

It was assumed that the carcinogenic potencies of *N*-Nitrosonornicotine (NNN), Nicotine-derived nitrosamine ketone (NNK), *N*-nitrosoanabasine (NAB) and *N*-nitrosoanatabine (NAT) are not significantly lower than that of NDMA (a risk assessment excluding NAT would arrive at the same conclusions) which probably results in an overestimation, but data are lacking to verify the extent of this overestimation. Increased incidences of tumors in the respiratory tract upon exposure to TSNAs for a bystander of e-cigarette vaping cannot be excluded for the ‘car’ scenario. For the ‘office’ scenario, an assessment of the risk cannot be made with sufficient certainty.

### 3.12. Copper

A tolerable concentration in air (TCA) for copper was set at 1 µg/m^3^ [20]. The exposure concentrations for both scenarios are below this limit value. It can be concluded that a risk for adverse health effects upon exposure to copper is not expected for bystanders of e-cigarette vaping.

### 3.13. Other Metals

The levels of vanadium, chromium, manganese, cobalt, nickel, zinc, arsenic, molybdenum, cadmium, tin, lead and uranium in the exhaled vapor were below the LOQ. Specific species of chromium, nickel and arsenic are carcinogenic, but it is unknown whether these forms are present in the exhaled air, since only total chromium, nickel and arsenic were measured. Therefore, no definite conclusions can be drawn about the carcinogenic risks. With regard to nickel and arsenic, assuming that the carcinogenic species of these metals are present, it can still be stated that the risk for cancer will be negligible for amounts below the LOQ. No conclusions can be drawn for chromium. 

For tin, no adequate toxicological data are available. For the remaining metals (vanadium, manganese, cobalt, zinc, molybdenum, cadmium, lead and uranium), it was concluded that amounts of these metals below their respective LOQs are not expected to induce adverse health effects based on the available toxicological information.

## 4. Discussion

We studied the exposure and health effects of bystanders to e-cigarette use, and found that bystanders may experience irritation to the upper respiratory tract and eyes, and systemic effects of nicotine, including an increased heart rate and higher systolic blood pressure. An increased risk of cancer could not be excluded. To the best of our knowledge, this represents the first toxicological risk assessment of e-cigarette vapor to bystanders. While health effects to bystanders are expected, the effects are relatively mild, even in extreme scenarios. Importantly while the levels of tobacco-specific nitrosamines in exhaled vapor are high enough that an elevated risk of cancer could not be excluded, only a limited number of e-liquids currently on the market contain significant quantities of TSNAs. The risks associated with these compounds could be avoided altogether by enforcing that e-liquids may not contain detectable amounts of TSNAs, in accordance with the European Tobacco Product Directive 2014/40/EU.

Hess et al. [21] recently conducted a systemic review of the health risks of passive exposure e-cigarette vapor. They found that e-cigarette vapor is likely to present a health risk to bystanders. In agreement with our assessment, irritation of the upper airways and eyes, and effects from nicotine are the main effects reported.

Studies published to date on second-hand vaping have employed three or fewer test subjects. Our study includes 17 test subjects. In view of the large variation in emissions between subjects, it appears that using larger numbers of subjects is important. Other than the number of puffs, we did not impose a vaping topography on the volunteers (i.e., they were free to ‘vape naturally’, in terms of puff length, volume and interval). This likely contributed to the variation observed in the emissions from different volunteers. Given the limited number of volunteers, it is very unlikely that the highest emissions we observed represent the worst case. We therefore expect that this leads to an underestimation of exposure.

We have included more than one e-cigarette/e-liquid combination in this study, to approximate real-world situations. The very large number of different products available to consumers prohibits comprehensive testing of all available types of devices and liquid. We have tested three different e-cigarette/e-liquid combinations. The products tested in this study (a small cig-a-like type device and a refillable clearomizer operated at 7.6 W) are relatively low-powered devices, compared to the range of products available. We therefore expect that our choice of products is likely to lead to an underestimation of exposure.

Our estimates of bystander exposure might be improved by accounting for the propagation of emissions, physical and chemical changes that occur in the vapor as it is suspended in the air, and deposition/desorption on solid surfaces. Particles may aggregate or evaporate, altering their dimensions, and consequently, their ability to reach the lower airways. Nicotine may react with nitrogen oxides in the air to form nitrosamines [22,23,24]. Components of the emissions may deposit on solid surfaces, and be released at a later time. These processes are highly dependent on the physical chemical properties of different compounds and the surfaces involved, and insufficient data is currently available to model these processes well.

Furthermore, we have sampled and analyzed only a single exhalation following each puff, because it contains the majority of the emissions, and have estimated the emissions exhaled in any following exhalations. This required us to make assumptions regarding breathing behavior. Sampling and analyzing the second and third exhalations would capture a larger fraction of the exhaled emissions, and possibly allow for a more accurate analysis of emissions without the need to model breathing behavior.

Finally, e-liquids contain components for which no adequate toxicological data is available, which includes the majority of flavor components and their decomposition products. Acute health effects have been experienced by e-cigarette users that might be attributable to specific flavor ingredients (4), and long-term effects may only be become apparent after several years. Many of the flavor compounds used in e-cigarettes are food additives that are Generally Recognized as Safe (GRAS), but not much is known about the toxicity of these components when they are inhaled. 

More information on the toxicity of inhaled flavor compounds is needed with some urgency, considering the number of different flavors available and the popularity of flavored e-cigarettes.

## 5. Conclusions

Vaping and breathing behavior, the characteristics of e-cigarettes and the dimensions and rate of ventilation of the room all have a large bearing on the concentrations of chemicals to which bystanders are exposed. In the ‘car’ scenario, we considered a situation in which two people vape in a confined, unventilated space. The level of exposure in this scenario will approximate the highest levels that should occur in real life. In this scenario, bystanders may experience irritation of the respiratory tract as a result of exposure to propylene glycol and glycerol. If nicotine-containing e-liquid is used, systemic effects of nicotine can occur, including palpitations and an increase of the systolic blood pressure, comparable to what may be expected from the intake of the amount of caffeine contained in 2 or 3 cups of coffee. Furthermore, due to the presence of TSNAs in some liquids, an increased risk of tumors cannot be excluded.

We believe the ‘office’ scenario to be more indicative of a typical level of exposure in real life. Health risks to bystanders were also identified in this scenario. While irritation of the respiratory tract is not expected, systemic effects of nicotine (palpitations, increased blood pressure) may be experienced.

Only a limited number of e-cigarettes and e-liquids were used in this study, and significant differences exist between products. A large variability in the exhaled amounts of chemicals was also observed between subjects using the same device and e-liquid, presumably due to differences in the individual vaping and breathing behavior of the volunteers. It would therefore be interesting to study the effects of vaping topology more extensively, as well as device design and e-liquid composition on the amount of exhaled chemicals in future studies.

### Regulatory Implications

Some of our findings are of relevance for regulatory purposes.

Firstly, the levels of tobacco-specific nitrosamines in exhaled vapor are high enough that an elevated risk of cancer could not be excluded. Considering that only a limited number of e-liquids currently on the market contain significant quantities of TSNAs, the risks associated with these compounds can be avoided altogether by enforcing that e-liquids may not contain detectable amounts of TSNAs, in accordance with the European Tobacco Product Directive 2014/40/EU.

Secondly, our study indicates that health risks to bystanders exist, but they could be considered relatively mild, especially considering the worst-case character of the investigated ‘car’ scenario. This result can be taken into consideration in policies for vaping in public spaces, together with other considerations such as the potential risk of increasing social renormalization of ‘smoking’.

Thirdly, insufficient toxicological data is currently available for many common flavor ingredients upon inhalation. While acute health effects experienced by e-cigarette users have been observed that might be attributable to specific flavor ingredients [4], long-term effects will only be become apparent after several years. Hungary has recently opted to ban the use of flavors in e-liquids. It is therefore important to continue to monitor the health effects of e-cigarette use.

## Figures and Tables

**Table 1:** E-cigarette/e-liquid combinations.

Designation	Specification	Flavor	Nicotine (mg/mL)	PG/Glycerol Ratio
A	Non-refillable ‘cig-a-like’ with rechargeable battery	tobacco	18	50/50
B	Refillable, dual-1.8 ohm bottom-coil	tobacco	18	50/50
	Battery: 3.7 V constant voltage, 1000 mAh			
C	Refillable, dual-1.8 ohm bottom-coil	tobacco	11	80/20
	battery: 3.7 V constant voltage, 1000 mAh			

**Table 2:** Average mass gain of the trapping device for individual subjects for control breath (‘control’) or e-cigarette vapor exhalations (‘exhaled vapor’).

Subject	Average Weight Gain of Filter/Cartridge Assembly (mg per Puff)	
	Control	Exhaled Vapor
1	1.76	1.41
2	1.31	3.31
3	0.87	5.97
4	1.12	3.63
5	3.27	5.04
6	2.63	6.07
7	0.00	0.94
8	3.70	11.00
9	4.04	5.39
10	0.84	5.60
11	3.89	5.34
12	2.68	5.93
13	1.72	3.55
14	0.38	1.21
15	0.53	0.83
16	1.52	4.00
17	1.28	2.96

**Table 3:** Chemical analysis of exhaled vapor. The column ‘total quantity’ lists average amounts recovered in the first exhaled breath after inhaling a puff.

	*n*	Total Quantity			
		Range		Median	
		Min	Max		
nicotine	17	<LOQ	2140	108	ng
humectants					
propylene glycol	17	<LOQ	127	<LOQ	μg
glycerol	17	<LOQ	<LOQ	<LOQ	μg
nitrosamines					
NNN	9	<LOQ	111	29	pg
NAT	9	<LOQ	40	14	pg
NAB	9	<LOQ	8	2	pg
NNK	9	<LOQ	71	15	pg
NDMA equivalent total TSNA	9	<LOQ	77	28	pg
aldehydes					
formaldehyde	4	<LOQ	<LOQ	<LOQ	ng
acetaldehyde	4	<LOQ	<LOQ	<LOQ	ng
acroleine	4	<LOQ	<LOQ	<LOQ	ng
metals					
arsenic	3	<LOQ	<LOQ	<LOQ	ng
molybdenum	3	<LOQ	<LOQ	<LOQ	ng
tin	3	<LOQ	<LOQ	<LOQ	ng
cadmium	3	<LOQ	<LOQ	<LOQ	ng
lead	3	<LOQ	<LOQ	<LOQ	ng
zinc	3	<LOQ	<LOQ	<LOQ	ng
copper	3	<LOQ	2.92	<LOQ	ng
nickel	3	<LOQ	<LOQ	<LOQ	ng
cobalt	3	<LOQ	<LOQ	<LOQ	ng
manganese	3	<LOQ	<LOQ	<LOQ	ng
chromium	3	<LOQ	<LOQ	<LOQ	ng
vanadium	3	<LOQ	<LOQ	<LOQ	ng
uranium	3	<LOQ	<LOQ	<LOQ	ng

‘Range’ lists the lowest and highest values observed. The median was calculated over all data, including samples with a value below the level of quantification. = (Averages were calculated over the number of exhaled breaths collected on each filter (5 for nicotine, propylene glycol and glycerol, 25 for the other analytes)).

**Table 4:** Parameters defining the two scenarios for which bystander exposure was estimated.

	Scenario 1	Scenario 2	
	(Car)	(Office)	
Number of persons vaping	2	1	
Puffing frequency	0.5	2	min^−1^
Total vaping time *	1	4	h
Volume of space	2	30	m^3^
Ventilation	0 (none)	0.5	h^−1^

* Exposure duration of the bystander is considered similar to total vaping time.

**Table 5:** Exposure concentrations and systemic doses.

		Scenario 1: Car		Scenario 2: Office	
	Amount Exhaled per Puff	Concentration for Assesment of Local Effects	Systemic Dose	Concentration for Assesment of Local Effects	Systemic Dose
propylene glycol	127 ug	9.5 mg/m^3^	0.087 mg/kg bw/d	2.16 mg/m^3^	0.032 mg/kg bw/d
nicotine	2.14 ug	0.16 mg/m^3^	0.00146 mg/kg bw/d	0.036 mg/m^3^	0.0005 mg/kg bw/d
TSNAs as “NDMA eq”	77 pg	5.8 ng/m^3^		1.31 ng/m^3^	
copper	2.92 ng	219 ng/m^3^		50 ng/m^3^	

The risk estimate for the tobacco specific nitrosamines was based on the assumption that risk of exposure to the individual nitrosamines can be equated to that of exposure to an equimolar concentration of NDMA. Therefore, the concentration of NDMA calculated to correspond with the exposure to the sum of the four tobacco-specific nitrosamines (TSNAs), is listed here as “TSNAs as NDMA eq”, even though NDMA itself was not measured, and is not expected to occur in the exhaled vapor.

**Table 6:** Margin of Exposure (MOE) for different chemicals and scenarios for the evaluation of local and systemic effects, calculated from points of departure (PoDs) and exposure (Supplementary Material 1 and Table 5, respectively).

	‘Car’ Scenario	‘Office’ Scenario	Endpoint
Local effects			
Propylene glycol	17	74 or 81 *	irritation of the upper respiratory tract
TSNAs	521	2297	tumors in upper respiratory tract
Systemic effects			
Propylene glycol	535	1475	reduced number of lymphocytes

* PoDs derived from two different studies (Supplementary Material 1).

## Supplementary Materials

The following are available online at https://www.mdpi.com/1660-4601/16/9/1525/s1, Supplementary Materials 1 and 2.

## Abbreviations

CAWI	computer assisted web interviewing
CFP	Cambridge filter pad
CX572	carboxen 572
DNPH	dinitrophenylhydrazine
EFSA	European Food Safety Authority
ICP-MS	inductively-coupled plasma mass spectrometry
LC-MSMS	liquid chromatography—tandem mass spectrometry
LOQ	limit of quantification
MREC	medical research and ethics committee
MOE	margin of exposure
MTBE	methyl-tert butyl ether
NAB	*N*-nitrosoanabasine
NAT	*N*′-nitrosoanatabine
NDMA	*N*-nitrosodimethylamine
NNK	4-(methylnitrosamino)-1-(3-pyridyl)-1-butanone
NNN	N-nitrosonornicotine
NOAEL	No-Observed-Adverse-Effect Level
PG	propylene glycol
PoD	point of departure
TCA	tolerable concentration in air
TSNA	tobacco-specific nitrosamine
