# Supplementary material for: The Health Risks of Electronic Cigarette Use to Bystanders
Source: Int J Environ Res Public Health. 2019 Apr 30;16(9):1525. doi: 10.3390/ijerph16091525 (PMC6539638; doi:10.3390/ijerph16091525)
Supplement: Supplementary file 1 [file ijerph-16-01525-s001.zip › ijerph-474630-suppl/ijerph-474630_supplement 1.docx]

# SUPPLEMENTARY MATERIAL 1

**Points of departure**

In order to obtain and select relevant information on possible adverse human health effects of the analyzed chemicals, reports and evaluations of (inter)nationally recognized organizations (among others WHO, US EPA, ATSDR, AEGL committee, the Health Council of the Netherlands) were used as primary sources.

The air concentrations for these scenarios are compared with human limit values (air concentrations) for chronic exposure for the general population for the purpose of risk assessment. These limit values are in general applicable to continuous exposure of 24h/d. *Air Quality Guidelines* as derived by the WHO are examples of such limit values and these will be used as first choice in the current evaluation (22).

## Propylene glycol

A subchronic inhalation study with rats showed that repeated exposure to propylene glycol in concentrations of 0, 160, 1000 and 2200 mg/m^3^ for 6h/d, 5d/week for 13 weeks resulted in effects on the respiratory tract (increased number of goblet cells) with a NOAEL of 160 mg/m^3^ and nasal hemorrhage with a LOAEL of 160 mg/m^3^ ([1] as described in [2]).

A human study was described in which a one minute inhalation exposure of healthy human volunteers (n=27) to 176 – 851 mg/m^3^ propylene glycol (geometric mean: 309 mg/m^3^) resulted in (subjectively reported) irritation of eyes and the upper respiratory tract. The lowest concentration of this exposure concentration range can be considered a LOAEL ([3] as described in [4]). Given the one-minute exposure period, this study was used as supportive.

Repeated inhalation exposure to propylene glycol resulted in the same rat study also in a reduced number of lymphocytes with a NOAEL of 160 mg/m^3^ ([1] as described in [2]); this is equivalent to 46.4 mg/kg bw/d^[[1]](#footnote-1)^. This value is used as PoD for the risk assessment of systemic effects.

In addition, a recommendation for maximum exposure levels of actors to propylene glycol via theatrical fog is also available. It was recommended that exposures to propylene glycol by actors should not exceed peak or ceiling concentrations of 40 mg/m^3^ [5].

## Nicotine

The available toxicological (inhalation) data for nicotine are very limited. An appropriate PoD for evaluating of a lifetime inhalation exposure is not available. A MOE-approach could therefore not be applied and a weight-of-evidence evaluation was applied.

#### Nicotine: local effects on the respiratory tract

Results of a two-year rat inhalation study show that repeated inhalation exposure of 0.5 mg/m^3^ nicotine during 103 weeks (20 h/d, 5d/wk) resulted in a small decrease of body weight. Macro- and microscopic evaluation did not show any treatment-related effect ([6] as decribed in [7]). The concentration of 0.5 mg/m^3^ was considered a NOAEL. Based on this study, it can however not be determined at which concentration effects are to be expected.

***Nicotine: systemic effects***

Hanssen *et al.* [8] conducted a human study in which 8 non-smoking volunteers inhaled nicotine solutions, administrating doses of 0, 0.4, 0.8 and 1.7 mg of nicotine over 5 minutes. A significant increase in heart rate and systolic blood pressure was observed, compared to vehicle-controls. The diastolic blood pressure did not change significantly.

#### Nicotine: effects on developing fetus

The potential effects of nicotine on the development of the fetus are investigated in some studies. These studies show that exposure to nicotine may result in a delayed development of the fetus (characterized by a reduced body weight, but also reduced fetal organ weights of various tissues such as brain, heart, lung). In addition, studies are available which show a reduced gestational period and abortions, and effects on male reproductive organs. However, some of these studies had some limitations as for example no data on maternal toxicity were presented in the developmental toxicity studies. Moreover, it should be noted that reproductive toxicity studies with exposure via the inhalation route are not described.

A study with rhesus monkeys shows that subcutaneous exposure (via a mini-osmotic pump) to nicotine in a dose of 1 mg/kg bw/d during gestation days 26-134 resulted in detectable nicotine levels in amniotic fluid, a 8% lowered fetal body weight, reduced body length and biparietal, and reduced fetal organ weights of heart, pancreas, adrenals, kidney and brain. Fetal lung weight and volume were reduced by 13% and 12%, respectively (not significant). Further, there were some indications that fetal lung development was changed in response to prenatal nicotine exposure. The lungs of offspring had hypoplasia and a reduced surface complexity of developing alveoli. Maternal body weight and food consumption was unchanged ([9] as described in [7]).

## Tobacco-specific nitrosamines (TSNAs)

The tobacco-specific nitrosamines NNK, NNN and NAB induce tumors in experimental animals; in general this considers lung tumors independent of the exposure route [10]. NNK and NNN are considered as genotoxic carcinogens and are classified by IARC as group 1 (‘carcinogenic to humans’) carcinogenic chemicals. NAB and NAT are classified by IARC as group 3 (‘not classifiable as to its carcinogenicity to humans’) carcinogenic chemicals [11]. In vitro genotoxicity studies showed that NNK has a similar mutagenic potency when compared to N-nitrosodimethylamine (NDMA), and a higher mutagenic potency when compared to NNN. Of these four mentioned tobacco specific nitrosamines, NNK has the highest carcinogenic potency, followed by NNN. NAB is considered a carcinogen with a moderate potency while hardly any evidence is available for NAT pointing towards potential carcinogenicity [10].

The available adequate toxicological (inhalation) data for the mentioned four tobacco-specific nitrosamines are quite limited. Therefore, inhalation data from the nitrosamine N-nitrosodimethylamine (NDMA) were used for the risk assessment of the tobacco-specific nitrosamines. Quantities of TSNA were converted to NDMA equivalents by calculating equimolar amounts (1 pg of NNN = 0.42 pg NDMA equivalents, 1 pg of NAT = 0.39 pg NDMA equivalents, 1 pg of NAB = 0.39 pg NDMA equivalents, 1 pg of NNK = 0.36 pg NDMA equivalents).

A rat inhalation study (n=36/group) with exposure to NDMA was selected to derive the PoD for risk assessment. Exposure to 0, 120, 600 and 3000 µg/m^3^ NDMA (4-5 h/d, 4 d/wk) during 207 days resulted primarily in tumors in the nasal cavity. These effects are considered relevant for the potency to induce respiratory tract tumors in humans. It is assumed that the 207 days refer to the number of exposure days, the total study period will therefore be one year. The tumor incidences for the nasal tumors were 0/36, 13/36, 31/36, 19/36, respectively ([12] as described in [13]). The results of this study were analyzed with a BMD-analysis and a BMDL10 was derived. A detailed description of the BMD-analysis and an overview of the results can be found in section 11.4 of our previous report [14] . The analysis resulted in a BMDL10 of 3 µg/m^3^.

1. Suber, R.L., et al., *Subchronic nose-only inhalation study of propylene glycol in Sprague-Dawley rats.* Food Chem Toxicol, 1989. **27**(9): p. 573-83.

2. ATSDR, *Toxicological profile for propylene glycol. Agency for Toxic Substances and Disease Registry, USA.* [*http://www.atsdr.cdc.gov/ToxProfiles/tp189.pdf*](http://www.atsdr.cdc.gov/ToxProfiles/tp189.pdf)*.* 1997.

3. Wieslander, G., D. Norback, and T. Lindgren, *Experimental exposure to propylene glycol mist in aviation emergency training: acute ocular and respiratory effects.* Occup Environ Med, 2001. **58**(10): p. 649-55.

4. Health Council of the Netherlands, *Propylene glycol (1,2-Propanediol). Health-based recommended occupational exposure limit. The Health Council of the Netherlands. No. 2007/02OSH.* [*http://www.gezondheidsraad.nl/sites/default/files/200702OSH.pdf*](http://www.gezondheidsraad.nl/sites/default/files/200702OSH.pdf)*.* 2007.

5. Toxnet, *Toxicology Data Network on Propylene Glycol. US National Library of Medicine.* [*http://toxnet.nlm.nih.gov/cgi-bin/sis/search/a?dbs+hsdb:@term+@DOCNO+174*](http://toxnet.nlm.nih.gov/cgi-bin/sis/search/a?dbs+hsdb:@term+@DOCNO+174)*.* 2016.

6. Waldum, H.L., et al., *Long-term effects of inhaled nicotine.* Life Sci, 1996. **58**(16): p. 1339-46.

7. Health Council of the Netherlands, *Nicotine (CAS no: 54-11-5). Health-based Reassessment of Administrative Occupational Exposure Limits (Revised version). Committee on Updating of Occupational Exposure Limits, a committee of the Health Council of the Netherlands. No. 2000/15OSH/105(R).* [*http://www.gezondheidsraad.nl/sites/default/files/0015105OSHR.PDF*](http://www.gezondheidsraad.nl/sites/default/files/0015105OSHR.PDF) 2005.

8. Hansson, L., et al., *Inhaled nicotine in humans: effect on the respiratory and cardiovascular systems.* J Appl Physiol (1985), 1994. **76**(6): p. 2420-7.

9. Sekhon, H.S., et al., *Prenatal nicotine increases pulmonary alpha7 nicotinic receptor expression and alters fetal lung development in monkeys.* J Clin Invest, 1999. **103**(5): p. 637-47.

10. Hoffmann, D., et al., *Tobacco-specific N-nitrosamines and Areca-derived N-nitrosamines: chemistry, biochemistry, carcinogenicity, and relevance to humans.* J Toxicol Environ Health, 1994. **41**(1): p. 1-52.

11. IARC, *IARC monographs on the Evaluation of Carcinogenic Risks to Humans. Volume 89. Smokeless Tobacco and Some Tobacco-specific N-nitrosamines. Lyon, France.* [*http://monographs.iarc.fr/ENG/recentpub/mono89.pdf*](http://monographs.iarc.fr/ENG/recentpub/mono89.pdf)*.* 2007.

12. Klein, R.G., et al., *Effects of long-term inhalation of N-nitrosodimethylamine in rats.* IARC Sci Publ, 1991(105): p. 322-8.

13. Health Council of the Netherlands, *N-Nitrosodimethylamine (NDMA). Health based calculated occupational cancer risk values. No. 1999/12OSH* [*http://www.gezondheidsraad.nl/sites/default/files/OSH12.PDF*](http://www.gezondheidsraad.nl/sites/default/files/OSH12.PDF)*.* 1999.

14. Visser, W., et al., *De gezondheidsrisico’s van e-sigaret gebruik*. 2015, Dutch National Institute for Public Health and the Environment (RIVM).

1. The inhalation exposure is converted to an equivalent systemic dose, based on a respiratory volume of 0.29 m^3^/kg bw for the rat for a 6-h exposure (ECHA, 2012) [↑](#footnote-ref-1)
