# Supplementary material for: The Health Risks of Electronic Cigarette Use to Bystanders
Source: Int J Environ Res Public Health. 2019 Apr 30;16(9):1525. doi: 10.3390/ijerph16091525 (PMC6539638; doi:10.3390/ijerph16091525)
Supplement: Supplementary file 1 [file ijerph-16-01525-s001.zip › ijerph-474630-suppl/ijerph-474630_supplement 2.docx]

# **SUPPLEMENTARY MATERIAL 2: Estimation of bystander** exposure

## Introduction

The risk assessment for the bystander of e-cigarette vaping is based on the chemical analyses of the exhaled breath (first exhalation after drawing a puff) of the volunteers vaping selected e-liquids (see main text).

The e-cigarette does not emit chemicals in between puffs. Chemicals are irregularly exhaled by the vaper and will cause a steady increase of the concentration in environmental air. The rate of increase will among others, depend on the vaping behavior of the vaper and the pulmonary retention of the chemical. On the other hand, the concentration will decrease e.g. as a result of inhalation and pulmonary deposition or absorption by the bystanders and/or ventilation of the room.

The actual source of exposure is the exhalation of chemicals by the vaper. Therefore, special effort has been made to estimate the total amount exhaled. Following a puff, the inhaled chemical will enter the alveoli (depending on the depth of breathing) and will be exhaled in the next few exhalations. The total amount exhaled will, among others depend on the (pulmonary) retention (*i.e.*, deposition and absorption) of the chemical. Since only the first exhaled breath following a puff was captured and analyzed, assumptions on breathing physiology were made to estimate the total amount exhaled from the amount in the first exhalation.

From the analyses of the first exhaled breath, the concentrations of the chemicals in indoor air were calculated for two specific predefined scenarios of e-cigarette use. The first scenario corresponds to a daily car trip during which a bystander is exposed to chemicals exhaled by two e-cigarette users. The second scenario resembles exposure of a bystander to chemicals exhaled by a single e-cigarette user during part of a working day in an office room (parameters defining these scenarios are provided in the main text).

## Human exposure

### Description of exposure scenarios

Two exposure scenarios were evaluated, described below. Table S2.1 presents the parameter settings of these two scenarios.

For both scenarios, the risk assessment is performed for the non-vaping person.

***Scenario 1 – car***

This scenario concerns two persons vaping in a car, with a child (the bystander) sitting in the same car being exposed to the chemicals exhaled by the two vapers. The total vaping time (and thus exposure duration of the bystander) is set at one hour resembling an everyday car trip. The puff frequency for both vapers is set at 0.5 min^-1^, which equals average vapers according to an earlier study we conducted (Visser, Geraets et al. 2015)

***Scenario 2 – office***

This scenario describes that one person is vaping in an office space while a second person is sharing the same office space and is exposed to the chemicals exhaled by the vaper. The total vaping time (and thus the exposure duration of the bystander) is considered to be four hours. The puffing frequency is set at 2 min^-1^. Both the vaping period and the puffing frequency equal an intensive (“heavy”) vaper according to an earlier study we conducted (Visser, Geraets et al. 2015) .

***Table S2.1*** *Parameter settings of the two predefined scenarios used for risk assessment.*

| ***Default parameter settings*** | |
| --- | --- |
| ***Scenario 1 - car*** |  |
| Number of persons vaping | 2 |
| Puffing frequency | 0.5 min^-1^ |
| Total vaping time * | 1 h |
| Volume car | 2 m^3^ |
| Ventilation | No |
|  |  |
| ***Scenario 2 - office*** |  |
| Number of persons vaping | 1 |
| Puffing frequency | 2 min^-1^ |
| Total vaping time * | 4 h |
| Volume office space | 30 m^3^ |
| Ventilation | Yes; 0.5 h^-1^ |

* exposure duration of the bystander is considered similar to total vaping time

Starting point for the exposure estimation in the two scenarios is the amount exhaled by the vaper(s) in the first exhalation following a puff. The highest amount measured in the breath exhaled by the volunteers is used in the calculations. From this amount, the total amount exhaled in the two scenarios is calculated taking into account that exhalation of the chemical may not have been complete in the first exhalation but may continue with subsequent exhalations. From the total amount exhaled the end concentration in the regarding room is calculated and used for risk assessment as a first approximation. In the evaluation step, when judging whether the MOE is sufficiently large, it is considered that in reality the concentration in air will steadily increase and the use of an end concentration will result in an overestimation of potential health risks.

## Estimation of the total amount of a chemical exhaled by a vaper following one puff

As described in the main text, volunteers were asked to repeatedly draw a puff and after each puff the exhaled air of the first exhalation was collected and analyzed for the presence of selected chemicals. The absolute amount of a chemical present in the first exhalation was measured and used to calculate air concentrations resulting from exhalation of inhaled e-cigarette vapor. This calculation requires additional information (*e.g.*, on pulmonary retention and vaping pattern) and assumptions were made when information is not available. Initially, straightforward worst-case estimations were made. If no health risks were anticipated under these conditions no further refinement was needed. If risks could not be excluded, a further refinement towards a more realistic exposure estimation was performed. Exposure estimation included the following steps.

The amount of a chemical exhaled by a vaper in the first exhalation after drawing a puff is, to a large extent, dependent on the fraction retained in the respiratory tract. The higher this fraction, the lower the amount of a chemical to be exhaled and thus the lower the bystander exposure. Retention includes deposition in the respiratory tract and absorption into the systemic circulation. Deposition may take place over the entire respiratory tract, depending on among others particle size and depth of inhalation. Gasses and vapors generally will enter the alveoli where gas exchange takes place. As a first worst-case approximation, it is assumed that retention only occurs in the deeper lungs and not in the so-called dead-space volume. This may underestimate the retention and thus overestimate the exposure of the bystander. For the risk assessment of systemic effects, it is further assumed that all deposited chemical is absorbed.

These assumptions mean that 100% retention via the inhalation route will in practice not occur since approximately 70% of the inspired volume reaches the alveoli where gas exchange takes place. The pulmonary retention is therefore maximally 0.7.

A distinction should be made between chemicals having local effects on the respiratory tract and chemicals having systemic effects upon pulmonary absorption. The exposure concentration (in a room) is the most important dose metric in case of local effects on the respiratory tract, whereas the internal systemic exposure (expressed as mg/kg bw) would be the dose metric of choice for evaluating systemic effects in the present evaluation.

The source of exposure for the bystander is the total amount of the chemical exhaled by the vaper which can be calculated from the total amount inhaled in one puff. However, only the amount exhaled in the first exhalation is measured and although the main fraction will be exhaled during the first exhalation, additional amounts of the inhaled chemical can be exhaled during the subsequent breathing cycles until the next puff. The first step then is to estimate the fraction of the amount inhaled that will be exhaled in the first exhalation. Next, the fraction of the inhaled amount that will be exhaled during the subsequent exhalations until the next puff, needs to be estimated. From these two estimates, the total amount of chemical exhaled following one puff can be estimated.

The amount exhaled will to a large extent depend on the pulmonary retention. In the present scenarios, a low retention would result in high air concentrations. Preferably, a chemical-specific value is to be used but often not available. Therefore, a pulmonary retention fraction of zero (which will seldom be the case in reality) is used as a first worst-case estimate for evaluation of local effects on the respiratory tract of the bystander since this would result in the largest amount exhaled. However, for systemic exposure the situation is different. Although assuming no pulmonary retention for the vaper will result in highest air concentrations for the bystander, at the same time this would result in a low internal systemic exposure for the bystander. Obviously the same retention estimate should be used both for the vaper as for the bystander. This implies that assessing systemic exposure for the bystander requires a different worst-case assumption for the pulmonary retention than for local effects. Table S2.2 shows the systemic exposure of the bystander as fraction of the inhaled amount of one puff by the vaper. It shows that the systemic exposure is potentially highest at a retention of 50%; this leads to an estimated fraction of 0.25 of the amount inhaled in one puff by the vaper that might become systemically available in the bystander. A pulmonary retention of 50% is therefore used as a first, worst-case estimate for evaluation of systemic effects of the bystander. It should be noted that a *pulmonary* retention of 50% corresponds to an *alveolar* retention of 0.71 (*i.e.,* 0.5/0.7).

*Table S2.2 Potential systemic exposure for the bystander (as fraction of the inhaled amount in one puff) as a function of the pulmonary retention.*

| **Pulmonary retention fraction for vaper and bystander** | **Fraction of amount in one puff exhaled by vaper** | **Potential systemic exposure for the bystander (as fraction of the inhaled amount in one puff)** |
| --- | --- | --- |
| 0.1 | 0.9 | 0.09 |
| 0.2 | 0.8 | 0.16 |
| 0.3 | 0.7 | 0.21 |
| 0.4 | 0.6 | 0.24 |
| ***0.5*** | ***0.5*** | ***0.25*** |
| 0.6 | 0.4 | 0.24 |
| 0.7 | 0.3 | 0.21 |

The basis for the calculations is the measured amount of chemical exhaled in the first exhalation after drawing a puff (as described in the materials and methods section of the main text). The fraction of the inhaled amount that is exhaled in the first exhalation can be estimated as follows, based on the method for smoking of tobacco cigarettes of Bos *et al*. (Bos, Hernández et al. 2012) with slight adaptations for vaping of e-cigarettes. Estimations are made separately for local effects on the respiratory tract and systemic effects as different estimates for pulmonary retention are required to obtain worst-case exposure estimates for the two types of effects.

With a default tidal volume at rest of 500 mL, 350 mL (*i.e.,* a fraction of 0.7) reaches the alveoli and mixes with 2000 mL functional residual capacity (FRC) leading to a total volume of 2350 mL (see table S2.3 for default parameter values). The remaining fraction of 0.3 (*i.e.,* corresponding to the dead space) does not reach the alveoli upon inhalation and the chemical present in this fraction is assumed to be completely exhaled.

*Evaluation of local effects on the respiratory tract:* For the fraction that reaches the alveoli (*i.e.*, 0.7), a worst-case default of no retention is assumed, meaning that this fraction of 0.7 will be completely exhaled. However, during the first exhalation only 350 mL (similar as the inhaled volume) of the total volume of 2350 mL is exhaled, *i.e.*, a fraction of 0.15 (*i.e.*, 350/2350). Combined with the exhaled dead space volume, a total fraction of 0.4 (*i.e.*, 0.3 + (0.15 x 0.7)) of the amount of a chemical present in one puff will be exhaled during the first exhalation after drawing that puff. **Thus, for chemicals inducing local pulmonary effects upon inhalation, the absolute amount of a chemical inhaled in one puff is calculated by dividing the amount present in the first exhalation following a puff (established by measurement, as described in the main text) by 0.4.**

*Evaluation of systemic effects*: For the fraction that reaches the alveoli (*i.e.*, 0.7), the alveolar retention fraction of 0.71 (*i.e*., equivalent to a pulmonary retention fraction of 0.5) applies, meaning that 0.29 of this fraction will be exhaled. However, during the first exhalation only 350 mL (similar as the inhaled volume) of the total volume of 2350 mL is exhaled, *i.e.*, a fraction of 0.15 (*i.e*., 350/2350). Thus from the amount that has reached the alveoli, a fraction of 0.044 (0.15 × 0.29) is exhaled with the first exhalation. Combined with the exhaled dead space volume, a total fraction of 0.33 (*i.e.*, 0.3 + (0.044 x 0.7)) of the amount of a chemical present in one puff will be exhaled during the first exhalation after drawing that puff. **Thus, for chemicals inducing systemic effects upon inhalation, the absolute amount of a chemical inhaled in one puff is calculated by dividing the amount present in the first exhalation following a puff (as described in the materials and methods section in the main text) by 0.33, which equals multiplication by 3.**

*Table S2.3 Default parameter values for the exposure estimation*

| **Parameter** | **Default values** |
| --- | --- |
| Tidal volume (rest) | 500 mL |
| Puff volume | 70 mL |
| Functional Residual Capacity (FRC) | 2000 mL |
| Breathing rate | 12 min^-1^ |
| Dead space | 30% |
| Pulmonary retention fraction* | 0.5 |
| Alveolar retention fraction* | 0.71 |

* values used for evaluation of systemic effects; for evaluation of local effects on respiratory tract a retention fraction of zero is assumed

However, exhalation of the chemical will continue in subsequent exhalations until the next puff is drawn. During each subsequent breathing cycle, clean air is inhaled and a fraction of 0.15 of the amount present in the alveoli will be exhaled as explained above. For scenario 2 with a puff frequency of 2 min^-1^ (highest frequency of the two scenarios), each puff is followed by 5 breathing cycles with clean air, assuming a breathing frequency of 12 min^-1^. Figure S2.1 depicts a simulation of the alveolar concentration, relative to the concentration immediately after puff drawing, in a scenario with a puff frequency of 2 min^-1^ and four different alveolar retention fractions: no retention, low retention (0.3), high retention (0.71) and full (100%) retention. As indicated in figure S2.1, in case of high retention (red curve) and full retention (green curve), the alveolar concentration has decreased to zero before drawing a new puff. Thus, starting from a pulmonary retention fraction of 0.5 (equivalent to an alveolar retention fraction of 0.71 (red curve in figure S2.1)) the total amount of a chemical that is inhaled in one puff, minus the fraction retained, will be exhaled completely before a new puff will be drawn. Thus the amount exhaled can be calculated from the total amount inhaled by adjustment for the fraction retained, *i.e.*, by multiplication with 0.5. Since the puff frequency in scenario 1 (*i.e.*, 0.5 min^-1^) is lower than in scenario 2, the same holds for scenario 1.

***Figure S2.1*** *Estimated relative alveolar concentration during an e-cigarette vaping session of 6 minute duration with a puff frequency of 2 min^-1^. Dark blue line (‘no retention’) corresponds to an alveolar retention fraction of zero; light blue corresponds to an alveolar retention fraction of 0.3; red line (‘high retention’) corresponds to an alveolar retention fraction of 0.71; green blue (‘full retention’) corresponds to an alveolar retention fraction of 1.*

### Calculating bystander exposure in the selected scenarios

The maximal final concentration in ambient air (*i.e.*, car or office space without ventilation) at the end of a vaping session by a specified number of persons during a given period of time can be calculated as:

**conc = (1-F_pulm,ret_) × A_puff_ × f × t × n / V**

conc = concentration of a chemical in room upon vaping (mg/m^3^)

F_pulm,ret_ = pulmonary retention fraction (zero or 0.5 for local and systemic effects, respectively)

A_puff_ = amount of a chemical inhaled with one puff (mg)

f = puff frequency (min^-1^)

t = vaping period (min)

n = number of persons vaping *

V = volume of room (m^3^)

** it is assumed that all persons vaping in the room use the same constant puff frequency*

The amount of a chemical inhaled with one puff (A_puff_) was calculated as:

**A_puff_ = A_exh-1_ / F_exh-1_**

A_puff_ = amount of a chemical inhaled with one puff (mg)

A_exh-1_ = amount of a chemical present in the first exhalation after drawing one puff (mg)

F_exh-1_ = fraction of the amount of a chemical present in one puff that will be exhaled during the first exhalation after drawing that puff (*i.e.*, for evaluating local pulmonary effects a fraction of 0.4 is assumed; for evaluating systemic effects a fraction of 0.33 is assumed)

The systemic (internal) exposure of the bystander is calculated as:

**D_syst_ = conc × F_pulm,ret_ × t × RV**

D_syst_ = systemic (absorbed) dose for the bystander (mg/kg bw/d)

conc = maximal final concentration in ambient air (*i.e.*, car or office space without ventilation) at the end of a vaping session by a specified number of persons during a given period of time (mg/m^3^)

t = vaping period (min) *

F_pulm,ret_ = pulmonary retention fraction

RV = respiratory volume (default: 0.2 l/min/kg bw for a 70 kg person according to ECHA (2012) (ECHA 2012), corresponding to 2 x10^-4^ m^3^/min/kg bw. As default for a child an RV of 0.5 L/min/kg bw (corresponding to 5 x10^-4^ m^3^/min/kg bw) was used (calculated as a worst-case default from a RV of 11 m^3^/24 h for a 4-6y old child based on (ECHA 2012))

** the exposure period is assumed to be similar to the vaping period for the selected exposure scenarios*

Ventilation was not included in scenario 1 (car; 1 h exposure). Scenario 2 did include ventilation, given that ventilation is obliged for office spaces. Table S2.4 shows the end concentrations for specific time periods up to 4 h. End concentrations are expressed relative to the end concentration at 1 h without ventilation. For the ‘office’ scenario, a ventilation rate corresponding to a replacement of the air of 0.5 office space per hour was assumed to be a realistic minimum.

*Table S2.4 End air concentrations upon vaping for a specific time period with or without ventilation. The concentrations are expressed relative to the concentration present after one hour vaping without ventilation (i.e., corresponding to y). End air concentrations were determined with ConsExpo (RIVM) (read from a plot) in a preset scenario where only the duration and total amount was adjusted. This value was compared with the end air concentration of the same scenario without ventilation to obtain the relative end air concentration at the given end time.*

| **vaping period** | **1h** | **2h** | **3h** | **4h** |
| --- | --- | --- | --- | --- |
| *without ventilation* | y | 2y | 3y | 4y |
| *with ventilation** | 0.75y | 1.2y | 1.5y | 1.7y |

* a ventilation rate corresponding to replacement of the air of 0.5 office space per hour is assumed as realistic minimum

## REFERENCES

Bos, P. M. J., et al. (2012). "Risk Assessment of Tobacco Additives and Smoke Components. RIVM letter report 340031001/2012. <http://www.rivm.nl/en/Library/Scientific/Reports/2012/oktober/Risk_assessment_of_tobacco_additives_and_smoke_components_a_method_proposal>."

ECHA (2012). "Guidance on information requirements and chemical safety assessment Chapter R.15: Consumer exposure estimation. Version 2.1. <https://echa.europa.eu/documents/10162/13632/information_requirements_r15_en.pdf>."

ECHA (2012). "Guidance on information requirements and chemical safety assessment. Chapter R.8: Characterisation of dose [concentration]-response for human health. Version 2.1, November 2012. ECHA, Helsinki, Finland."

RIVM "Consexpo http://www.rivm.nl/en/Topics/C/ConsExpo."

Visser, W., et al. (2015). De gezondheidsrisico’s van e-sigaret gebruik, Dutch National Institute for Public Health and the Environment (RIVM).
